# A new automated national register-based surveillance system for outbreaks in long-term care facilities in Norway detected three times more severe acute respiratory coronavirus virus 2 (SARS-CoV-2) clusters than traditional methods

**DOI:** 10.1017/ice.2022.297

**Published:** 2023-09

**Authors:** Kirsten Gravningen, Petter Nymark, Torgeir B. Wyller, Oliver Kacelnik

**Affiliations:** 1 Department of Infection Prevention and Preparedness, Norwegian Institute of Public Health (NIPH), Oslo, Norway; 2 Department of Microbiology and Infection Control, Akershus University Hospital, Nordbyhagen, Norway; 3 Institute of Clinical Medicine, University of Oslo, Oslo, Norway; 4 Department of Geriatric Medicine, Oslo University Hospital, Oslo, Norway

## Abstract

**Objective:**

To develop and test a new automated surveillance system that can detect, define and characterize infection clusters, including coronavirus disease 2019 (COVID-19), in long-term care facilities (LTCFs) in Norway by combining existing national register data.

**Background:**

The numerous outbreaks in LTCFs during the COVID-19 pandemic highlighted the need for accurate and timely outbreak surveillance. As traditional methods were inadequate, we used severe acute respiratory coronavirus virus 2 (SARS-CoV-2) as a model to test automated surveillance.

**Methods:**

We conducted a nationwide study using data from the Norwegian preparedness register (Beredt C19) and defined the study population as an open cohort from January 2020 to December 2021. We analyzed clusters (≥3 individuals with positive SARS-CoV-2 test ≤14 days) by 4-month periods including cluster size, duration and composition, and residents’ mortality associated with clusters.

**Results:**

The study population included 173,907 individuals; 78% employees and 22% residents. Clusters were detected in 427 (43%) of 993 LTCFs. The median cluster size was 4–8 individuals (maximum, 50) by 4-month periods, with a median duration of 9–17 days. Employees represented 60%–82% of cases in clusters and were index cases in 60%–90%. In the last 4-month period of 2020, we detected 107 clusters (915 cases) versus 428 clusters (2,998 cases) in the last period of 2021. The 14-day all-cause mortality rate was higher in resident cases from the clusters. Varying the cluster definitions changed the number of clusters.

**Conclusion:**

Automated national surveillance for SARS-CoV-2 clusters in LTCFs is possible based on existing data sources and provides near real-time detailed information on size, duration, and composition of clusters. Thus, this system can assist in early outbreak detection and improve surveillance.

The large number of outbreaks in long-term care facilities (LTCFs) during the coronavirus disease 2019 (COVID-19) pandemic with severe outcomes among elderly, frail residents has highlighted the need for more accurate and timely infection surveillance.^1–3^ The main limitations of traditional surveillance are under-ascertainment, underreporting, and lack of timeliness and completeness of surveillance data.^4^ Outbreaks frequently also involve staff, leading to problems in the delivery of care and increased costs.^1^ An effective outbreak surveillance system for LTCFs must integrate information on both staff and residents. Such surveillance will apply equally to agents other than severe acute respiratory coronavirus virus 2 (SARS-CoV-2).

Many countries, including Norway, published specific infection prevention and control (IPC) advice for outbreaks in LTCFs.^5,6^ To calibrate these measures against possible harmful effects on residents’ functional level and quality of life, it is vital to have accurate and timely information on ongoing outbreaks. Although it is mandatory to notify the Norwegian Institute of Public Health (NIPH) of all potential outbreaks in healthcare institutions through the web-based notification system Vesuv,^7^ the data are often incomplete and labor intensive for local staff to collate. In 2020–2021, a total of 227 SARS-CoV-2 outbreaks in LTCFs including 2,729 cases, were notified through Vesuv.^8,9^ The ratio of residents versus employees was reported in 35%–57% of outbreaks and 42%–46% of the cases were among staff.

In Norway, ∼35,000 people reside in >900 LTCFs. The median age of residents is >85 years.^10^ During the pandemic, guidelines from national authorities recommended that LTCF residents with COVID-19 should not be routinely transferred to hospitals and staff should only be employed in 1 LTCF at a time.^11^

In June 2020, a new real-time population-wide Norwegian preparedness register for COVID-19, Beredt C19, was established as part of the legally mandated responsibilities of the NIPH during epidemics.^12^ It combines individual-level data from multiple existing sources to provide authorities with updated knowledge about the pandemic.

Infection surveillance in healthcare is changing from manual data collection by clinical staff to digital systems using unique person identifiers with privacy safeguards that can merge existing individual-level data from various sources.^3^

We sought to determine whether we could use Beredt C19 to develop an automated and more complete system for detecting, defining, and characterizing outbreaks in LTCFs. We evaluated the use of existing data in Beredt C19 (1) to detect and analyze the size, duration, and composition of SARS-CoV-2 clusters among residents and staff in LTCFs in near real-time, (2) to examine residents’ mortality associated with clusters and how the virus variant impacted on these clusters, and (3) to provide recommendations for future monitoring of outbreaks in LTCFs.

## Methods

### Data sources

We extracted data from Beredt C19, a national preparedness register containing deidentified individual-level data for the entire Norwegian population from January 2020 as described elsewhere.^12^ For this study, we merged data from 6 registers (Table 1).

**Table 1.** The Six Registers from Beredt C19 Used in the Study

Register	Description of Register	Data Used
The State Register of Employers and Employees (Aa Register)	Data on all employment relationships in Norway, excluding freelancers and the self-employed: https://www.nav.no/en/home/employers/nav-state-register-of-employers-and-employees	Occupational code, occupation, contract start date, contract end date, organizational number
Cause of Death Registry (DÅR)	Individual level information about causes of death: https://www.fhi.no/en/hn/health-registries/cause-of-death-registry/	Date of death
Norwegian Registry for Primary Health Care (KPR)	Database for residents in LTFCs: https://helsedata.no/en/forvaltere/norwegian-directorate-of-health/norwegian-registry-for-primary-health-care-kpr/?	Organization name and number, type of institution, type of stay, date of admission, date of discharge or death
Norwegian Surveillance System for Communicable Diseases (MSIS) including results from the MSIS Laboratory Database	Laboratory database with information about samples and analysis for COVID-19 and influenza: https://www.ehelse.no/prosjekt/msis-databasen	Positive SARS-CoV-2 PCR test results: test date Virus variant: test date, virus variant sequencing result
National Population Register (NPR)	Information about the total population in Norway: https://www.skatteetaten.no/en/person/national-registry/	Sex, county number, birthdate
Norwegian Immunization Registry (SYSVAK)	Register for immunization and vaccination status: https://www.fhi.no/en/hn/health-registries/norwegian-immunisation-registry-sysvak/	Date of consultation, vaccine code

According to a legal mandate, all confirmed SARS-CoV-2 test results and virus sequencing results are automatically transferred from the microbiology laboratories to the national MSIS laboratory database.^13^ The NIPH has established a computer algorithm that will include a positive test result as soon as it is available (updated every 24 hours); thus, a new case will be included in ∼3–4 days from the test date.

### Study population and period

We defined the study population as an open cohort from January 1, 2020, to December 31, 2021. The resident population was selected from NPR (Table 1). The person had to be alive and registered as a long-term or temporary resident in an LTCF at some point during 2020–2021. Using this definition, the open cohort population of residents was 37,690 distributed across 993 facilities. The employee population was selected from the Aa Register based on registered employment in 1 of 993 facilities during 2020–2021. Altogether, 136,217 employees were included in the open cohort, for a study population of 173,907. We have also described both the study population (individual level) and the LTCFs (institutional level) at study start, using point prevalence estimates on January 1, 2020.

The study period was divided into 4-month periods. For each period, we analyzed cluster characteristics: the index case, size, number, composition, and duration. We calculated vaccine coverage for each period by linking the study population with SYSVAK data. For a second vaccine dose to be valid, it had to be 19 days after the first dose. Two doses were considered full vaccination during this period.

### Variable definitions

We defined a SARS-CoV-2 case as a person with a confirmed positive SARS-CoV-2 RT-PCR test from MSIS. We defined a SARS-CoV-2 cluster as ≥3 individuals (residents and employees) with SARS-CoV-2–positive test results from MSIS occurring within 14 days of each other that were linked to the same LTCF institutional number in the study period.^14^ Clusters could include any combination of residents and employees. An individual could have multiple positive tests and be part of multiple clusters occurring on different dates. The duration of a cluster was the number of days from date of the first positive SARS-CoV-2 test to the date of the last positive test in a cluster. The 14-day all-cause mortality included any instance of death by any cause within 14 days of a positive SARS-CoV-2 test. The 14-day all-cause mortality rate was the number of 14-day all-cause fatalities divided by the number of SARS-CoV-2 cases within a period or cluster. For example, 2 deaths within a cluster with 11 cases would yield a rate of 18%.

Because LTCF residents with COVID-19 were not generally transferred to hospitals during the pandemic, it was not feasible to use hospital admission as a surrogate marker for infection severity. Instead, we calculated the all-cause mortality rate within 14 days of a positive SARS-CoV-2 test among residents. We compared the 14-day all-cause mortality rate in clusters to the same measure in all LTCF-residents in Norway who tested positive in the study period. Although SARS-CoV-2 sequencing was not done for all cases, we used the available sequencing results to calculate the dominant virus variant per week among residents.

### Data analyses

We performed descriptive analyses and created figures using R version 4.0.2 software (R Foundation for Statistical Computing, Vienna, Austria). The process of detecting clusters and extracting information about them was automated using R, called automated cluster detection.

### Ethics statement

Beredt C19 was established under Section 2–4 of the Health Preparedness Act.^15^ The NIPH has conducted a data protection impact assessment of the register. The work included in this study was conducted as part of the mandated work of NIPH. All the data used in this study were stored on secure servers at the NIPH where only the authors employed at the NIPH had access. Details of data protection measures in Beredt C19 are described elsewhere.^12^

## Results

The study population at the start of the study on January 1, 2020, included 122,682 individuals in 993 LTCFs in Norway, of whom 79% were employees (88% women) (Table 2). The median age of residents was 88 years and more than two-thirds were women. The mean number of residents in the LTCFs was 34 and the maximum was 244. Furthermore, for a surveillance system to reflect the reality of LTCFs, it must include information on both residents and the staff who work there at any given time.

**Table 2.** Characteristics of 122,682 Individuals in the Study Population and the 993 Long-Term Care Facilities (LTCFs) at the Institutional Level^a^ on January 1, 2020

Characteristic	Residents, No. (%)	Employees, No. (%)
**Study population (individuals)**		
Total no.^b^	32,028 (21.1)	90,654 (78.9)
Gender, female	21,971 (68.6)	80,047 (88.3)
Mean age (SD)	86.5 (8.4)	41.7 (15.0)
Median age (IQR)	88 (81-93)	41 (28-54)
**LTCFs (institutions)**		
Mean no. (SD)	34.0 (29.7)	88.4 (62.0)
Median no. (IQR)	27 (14-47)	73 (45-119)
Maximum no.	244	510

Note. IQR, interquartile range; SD, standard deviation.

a Bottom 3 rows.

b Row percentage.

### Clusters

The main body of this study concerns data collected during the first 2 years of the pandemic. Table 3 divides these 2 years into 4-month periods.

**Table 3.** SARS-CoV-2 Cases and Clusters With ≥3 cases in 4-Month Periods in the Study Population in Long-Term Care Facilities (LTCFs) from March 2020 to December 2021

	Cases		Clusters					
Year and 4-Month Period	Total, No.^a^	Employees, No. (%)	Employee as Index, No. (%)	Total, No.	Size, Mean (SD)	Size, Median	Size, Max	Median Duration, Median Days (SD)
2020 1^b^	441	263 (59.6)	23 (60.0)	39	11.3 (10.2)	8	50	17.0 (13.5–22.0)
2020 2	28	21 (75.0)	5 (83.3)	6	4.7 (2.0)	4	8	8.5 (3.8–11.0)
2020 3	915	644 (70.4)	91 (85.0)	107	8.6 (7.7)	5	48	15.0 (9.0–22.0)
2021 1	649	523 (80.6)	89 (86.4)	103	6.3 (5.8)	4	42	14.0 (8.3–21.8)
2021 2	497	390 (78.5)	74 (87.1)	85	5.8 (5.3)	4	40	14.0 (7.0–22.0)
2021 3	2,998	2,464 (82.2)	383 (89.5)	428	7.0 (6.1)	5	45	17.0 (10.0–28.0)

Note. SD, standard deviation; max, maximum.

a Both residents and employees.

b The first SARS-CoV-2 cases were detected in week 10, 2020. The period 2020 1 was ∼2 months.

Clusters were detected in 427 (43%) of 993 LTCFs. The number of clusters varied, with relatively lower mean numbers in the summers of 2020 and 2021 and increasing numbers in the winter. In the last 4-month period of 2021, with the predominance of the SARS-CoV-2 δ (delta) variant, we detected 4 times as many clusters as in the last period of 2020 with predominance of the α (alpha) variant. The median size of the clusters was 8 in the beginning of 2020 and 5 toward the end of 2021. The mean and maximum size of the clusters also remained even, apart from during the second 4-month period of 2020. The median duration of clusters was 14–17 days, apart from the second period of 2020, when it was 8.5 days.

Employees made up 79% of our study population under surveillance at study start January 1, 2020 (Table 2). Employees represented between 60% and 82% of cases depending on the period (Table 3). We found no obvious overrepresentation of either staff or residents in the clusters. We also identified the index case for any cluster. The percentage of clusters for which an employee (rather than a resident) was the index (likely primary) case increased from 60% in the first period of 2020 to ∼85% for the rest of the study. The proportion of women in both the resident and employee clusters followed the proportion in the study population.

### Effect of modifying the cluster parameters

We tested the effect of changing the minimum requirement for cases to be included as a cluster from 3 to 5 or 10 and the effect of changing the maximum number of days between cases from 14 to 10. Increasing the minimum number of cases to 5 or 10 reduced the total number of clusters from 768 to 392 or 132 and changing the number of LTCFs affected reduced the total from 427 to 275 or 119. Moving from 14 days between cases to 10 days reduced the number of clusters from 768 to 702 and reduced the number of LTCFs affected from 427 to 388.

### Vaccination in LTCFs

Starting December 27, 2020, LTCF residents received vaccination against COVID-19, whereas employees were increasingly prioritized for vaccination from early January 2021. In the first 4-month period of 2021, residents attained vaccine coverage of 84%, but coverage was 36% for residents in the clusters (Table 4). Attainment of high vaccine coverage was slower for employees; they did not reach 80% until the second 4-month period of 2021. The coverage within the clusters for employees in the same period was 44%. In the last 4-month period of 2021, when everyone had the opportunity for full vaccination, the difference in vaccine coverage for all residents versus residents in clusters was 5%, whereas this difference was 12% (*P* < .001) between all employees and employees in clusters.

**Table 4.** Vaccination Coverage (2 doses) of the Study Population (n = 173,907) Divided into Residents and Employees and Vaccine Coverage Within the clusters from January 1, 2020, to December 1, 2021

Year and 4-Month Period(s)	Vaccinated Residents, %	Vaccinated Residents in Clusters, %	Vaccinated Employees, %	Vaccinated Employees in Clusters, %
2020 1–3	0	0	0	0
2021 1	83.9	35.7	18.7	2.3
2021 2	90.8	89.7	79.7	43.8
2021 3	94.8	89.7	90.1	78.6

### Cluster visualization

**Fig. 1.**
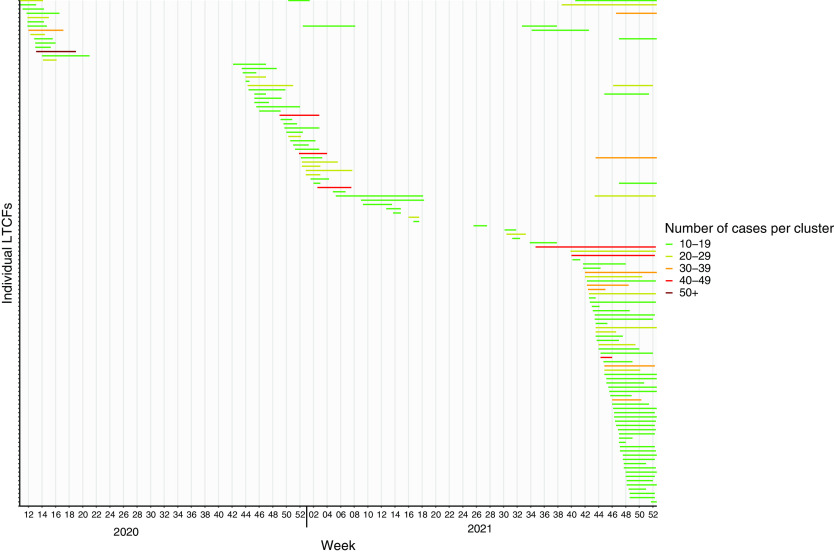
Clusters including ≥10 cases of SARS-CoV-2 in 119 LTCFs in Norway in 2020–2021. The horizontal lines show cluster duration and color indicates the number of cases in each cluster.

Figure 1 is a visualization of 132 clusters detected with ≥10 cases in 119 LTCFs, the first clusters occurred in week 10 (March 2020).

The figure demonstrates the grouping in terms of time of all the clusters with 10 or more cases in LTCFs. Although the number of clusters varied, the relative contribution of different sized clusters was similar. Furthermore, the number of clusters had already increased greatly through the predominance of the SARS-CoV-2 δ (delta) variant (weeks 31–52 of 2021) and prior to the ο (omicron) variant being dominant in the LTCF population in Norway. This figure can be automated and updated daily. Lastly, it illustrates the long duration of many of these clusters in LTCFs.

### Analysis of LTCF residents

**Fig. 2.**
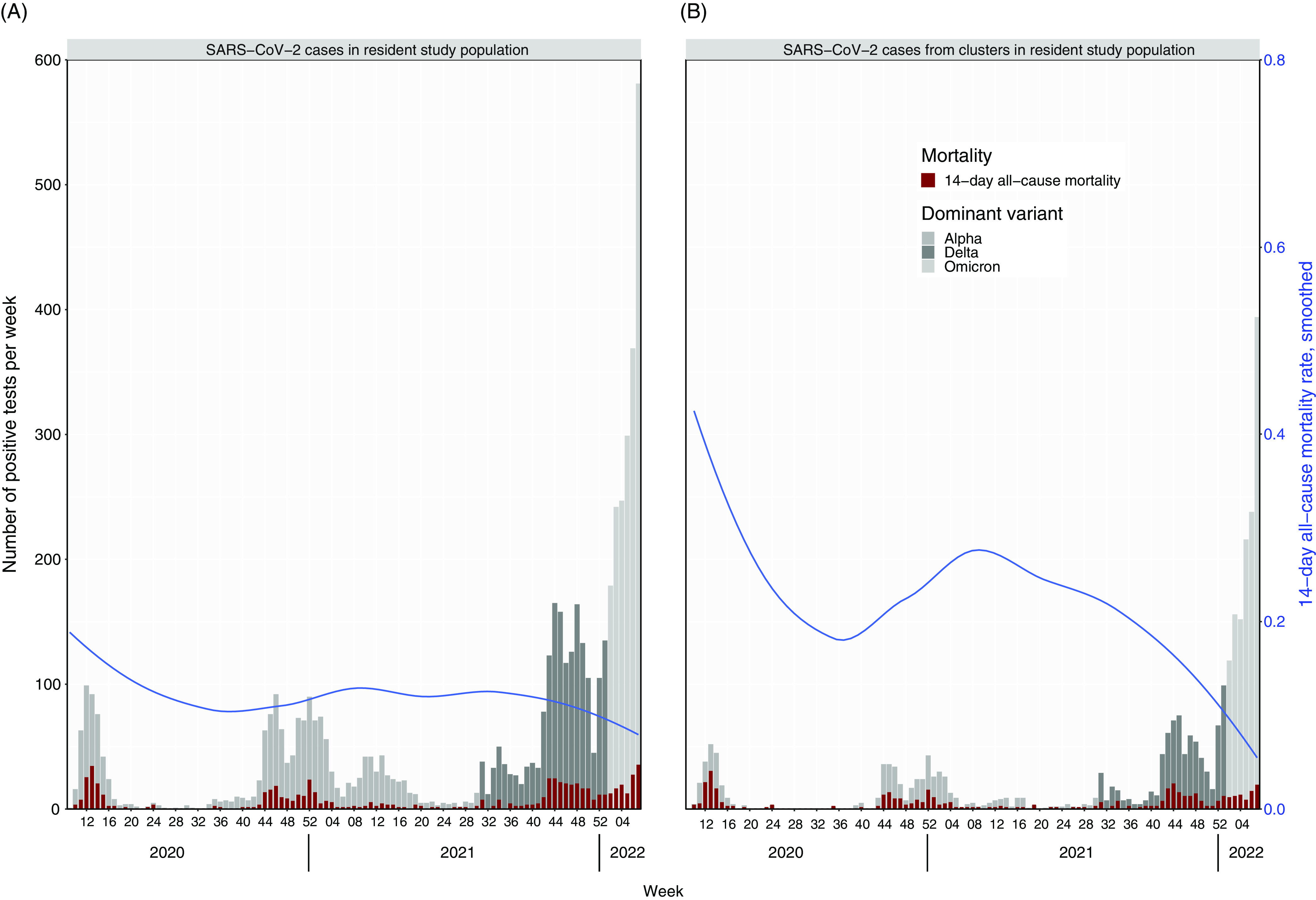
(A) Bar plot showing number of SARS-CoV-2 cases per week from March 2, 2020, to February 21, 2022, by the dominant virus variant for the resident study population. The blue smoothed-line graph shows the 14-day all-cause mortality rate over time. (B) The same data are shown for SARS-CoV-2 cases that were part of the clusters with ≥3 cases.

To contextualize surveillance information on clusters, we designed a system that would set up our clusters to consider all SARS-CoV-2 infections and would additionally include the type of virus variant in the resident study population and the 14-day all-cause mortality rate. We present findings beyond the main study period through February 21, 2022, including the introduction and dominance of the ο (omicron) variant. This part of the analysis included clusters with ≥3 cases and excluded all data on employees. In Figure 2, panel A shows the number of SARS-CoV-2 cases for the resident study population and panel B shows the same data for residents who were part of the clusters.

The pattern of positive SARS-CoV-2 tests in our clusters follows closely the results from the resident study population. Cases in the clusters had higher overall 14-day all-cause mortality rate early in the pandemic. Overall, the 14-day all-cause mortality rate was markedly higher among SARS-CoV-2 cases who were part of identified clusters than among cases in the general resident study population. However, in both the clusters and the resident study population we observed a decrease in the mortality rate over the course of the pandemic. This decrease was also pronounced in the period when SARS-CoV-2 ο (omicron) variant dominated, when the total number of cases was much higher.

## Discussion

Using COVID-19 as a model, we designed and implemented a system for automated national surveillance of infectious disease clusters in LTCFs in Norway, many of which were previously unreported. Using only data that were already available, we followed and analyzed COVID-19 clusters in LTCFs during 2020–2021. We tracked the size, duration, and composition of clusters as well as the index case for each cluster. Using denominator data representative of the LTCF population in Norway, we calculated the all-cause 14 day-mortality rate over time and found that it was associated with the predominant virus variant and was higher among resident cases who were part of the clusters than in cases from the resident study population.

Our new surveillance system has the potential to tackle problems such as underreporting, lack of timeliness, and incompleteness of data. The system detected a 3-fold increase in clusters with ≥3 cases and twice the number of cluster cases compared to our traditional method.^8^ Data on the index case, cluster composition, and vaccination coverage were complete and updated in the new system, but very few notifications in Vesuv were updated by LTCFs after the outbreak was over. Consequently, the traditional method underestimates the number, size, and duration of SARS-CoV-2 outbreaks in LTCFs. This underestimation may lead to a biased evaluation of the effect of the ICP measures used, which is an important factor in all countries implementing control measures.

Long outbreaks lead to increased staff absences, and increased restrictions should be implemented for prolonged periods every time an outbreak is suspected.^16^ Continuous, timely surveillance with cluster detection and the inclusion of both residents and employees enables early outbreak identification and containment, which can decrease the spread within and across facilities and can also reduce outbreak duration, staff absence, and resident morbidity and mortality. The higher all-cause mortality rate in clusters may have been caused by higher viral loads in infected individuals during outbreaks^14,17^ and/or by the reduced quality of care due to significant numbers of staff being absent during outbreaks.

Because our system is based on existing data recorded for other purposes, it does not require extra activity from the LTCFs. Combining laboratory data with individual data on residents and employees in each LTCF, their vaccine status, and residents’ date of death, we were able to automate the rapid detection of SARS-CoV-2 clusters in LTCFs in Norway. The surveillance system can also be used for other agents such as influenza virus.^18^ Because Beredt C19 includes population-based, deidentified, individual-level data, the registry has stable denominators and is considered representative of the LTCF population in Norway over time. Lastly, the visualization of cluster surveillance may contribute to increased awareness and should be further developed.

When the minimum requirement for cases to be included as a cluster was changed from 3 to 5 (a 60% increase), the number of clusters was reduced by ∼50%. However, moving from 14 days between cases to 10 days reduced the number of clusters by <10%. This new system is adaptable enough to react to changes in background noise (eg, community infection rates) and incubation times (eg, new SARS-CoV-2 variants).^19^ Furthermore, we can easily adjust the parameters for different agents that may be under surveillance.

In Europe, the lack of special surveillance systems and the differences in testing strategies and capacities among countries during the pandemic may have led to underdetection and underreporting of cases in LTCFs.^4^ The European Centre for Disease Prevention and control (ECDC) recommends a national comprehensive and mandatory LTCF-based surveillance system with cumulative or weekly reporting of cases among residents and staff. The development of electronic reporting through national platforms is considered crucial. Our new system for cluster detection takes this recommendation a step further.

### Strengths and limitations

The main strength of our study is the potential for accuracy and timeliness of the new nationwide surveillance system. We included both residents and staff, thereby providing the full picture at little extra cost. The study depended on mandatory, population-based registries, personal identifiers for follow-up of individuals within registries, linkage between registries, and between registries and other data sources.^20^ It required test results that may have been influenced by local and national testing strategies. Virus variant surveillance depends on genomic sequencing capacity, costs, and which analyses are requested by clinicians. SARS-CoV-2 data are very accurate for the first 2 years of the pandemic due to extensive and free PCR testing. Recent changes in recommendations that PCR be mainly used for clinical cases and not for screening make surveillance data potentially less complete. Furthermore, any delay in the data delivery or changes in data structure will affect the timeliness of analyses. Our method captured clusters on the institutional level and not by ward because this information was not included in the national registers. The clusters may reflect potential outbreaks more accurately in small LTCFs than in larger institutions with a high number of residents and departments; thus, a cutoff for clusters at ≥3 cases may have been too low in large facilities. Lastly, our data were censored at the end of 2022, so the size and duration of outbreaks that continued into 2023 may have been underestimated. This would not be a problem in an established surveillance system.

Future studies should explore how the cluster definition can be adjusted according to the size of the LTCFs, community infection rates, and incubation times of different agents. A system for reporting identification of clusters back to the LTCFs should be established to provide opportunities to use the outcome data from automated surveillance locally.^21^ Ideally, the implementation of the new system should be linked to scientific evaluations to efficiently collect the necessary information and to examine how the identified clusters correspond to real outbreaks.

We recommend the implementation of automated register-based surveillance of clusters in LTCFs wherever possible prior to the next winter respiratory virus wave in parallel with the development of systems to disseminate this information to individual LTCFs.
